# Supplementary material for: Barium bioaccumulation by bacterial biofilms and implications for Ba cycling and use of Ba proxies
Source: Nat Commun. 2018 Apr 24;9:1619. doi: 10.1038/s41467-018-04069-z (PMC5915594; doi:10.1038/s41467-018-04069-z)
Supplement: Supplementary file 1 — Supplementary Information [file 41467_2018_4069_MOESM1_ESM.pdf]

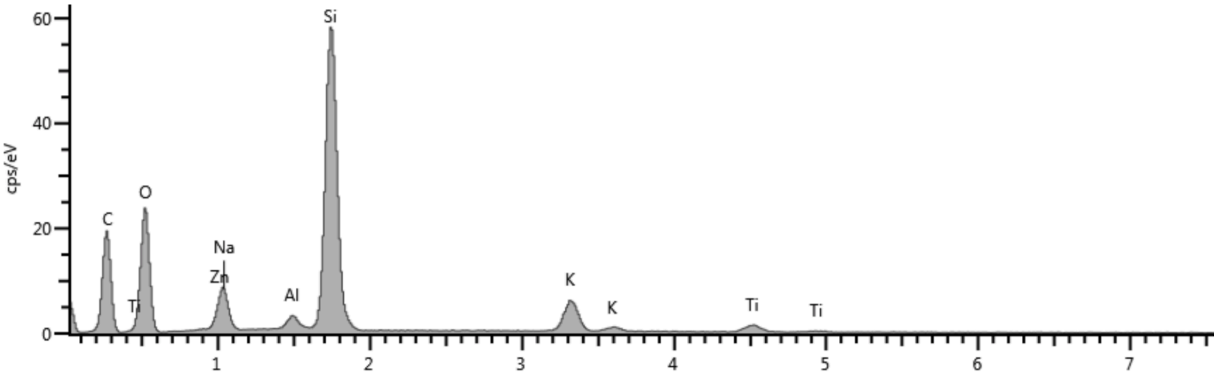

Supplementary Fig. 1. SEM-EDX spectrum showing the composition of the glass coverslips used for the experiments.

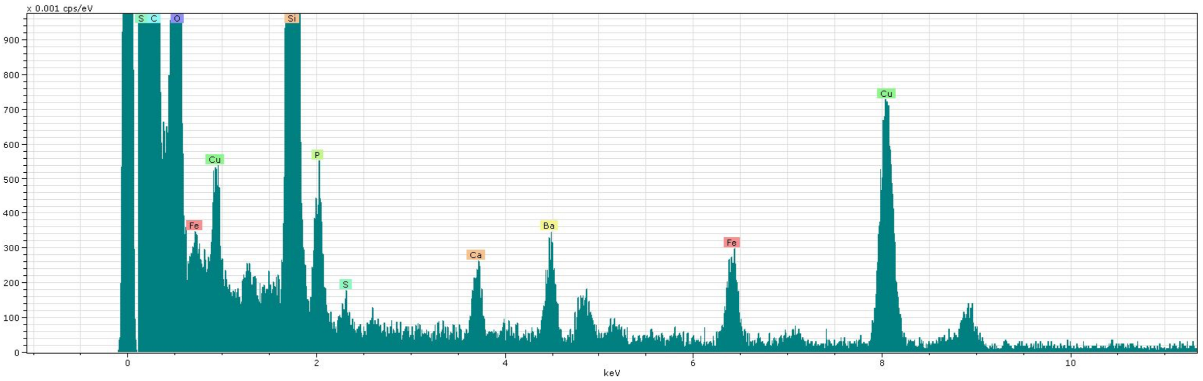

Supplementary Fig. 2. TEM-EDX spectrum showing the composition of the Ba precipitates (Fig. 4b).

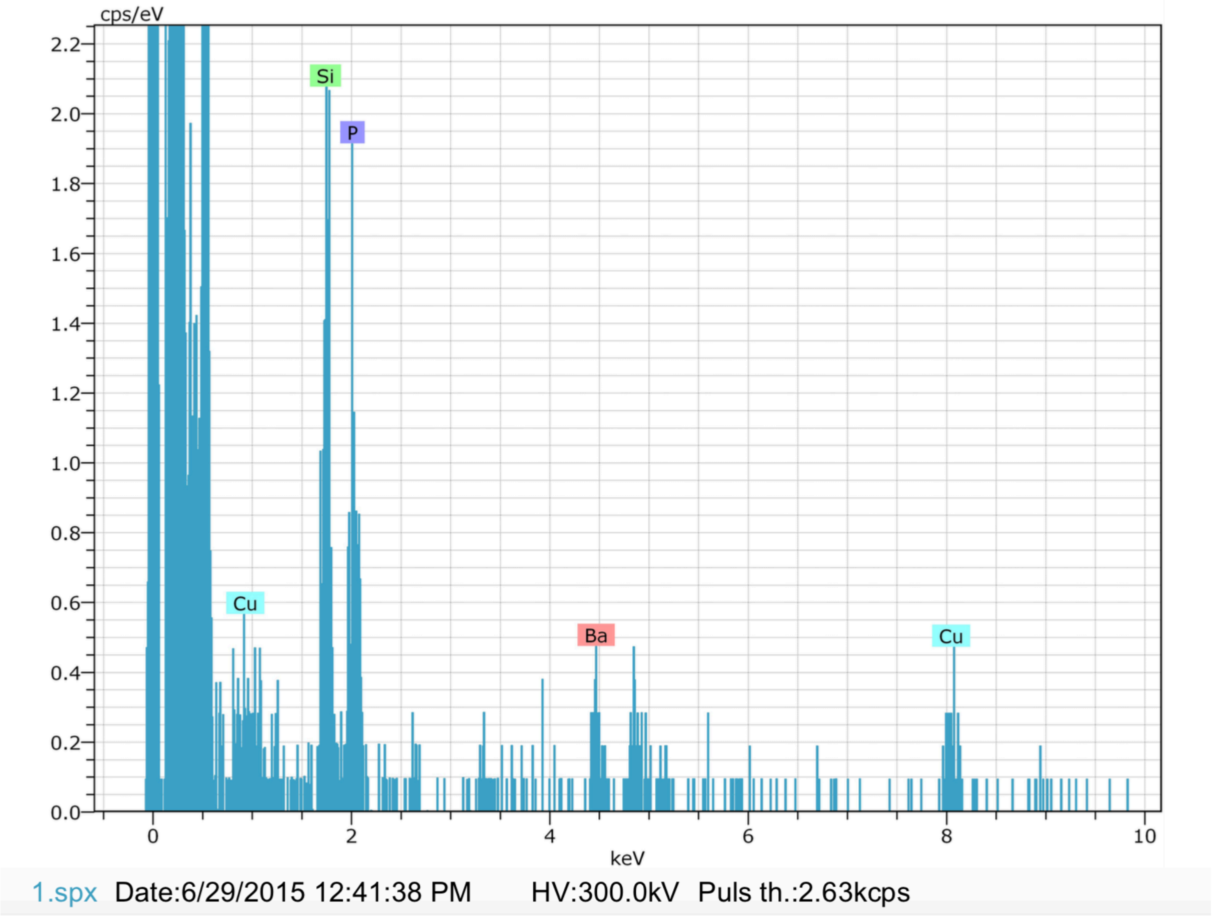

Supplementary Fig. 3. TEM-EDX spectrum showing the composition of a likely polyphosphate grain in bacterial cells (Fig. 3c).

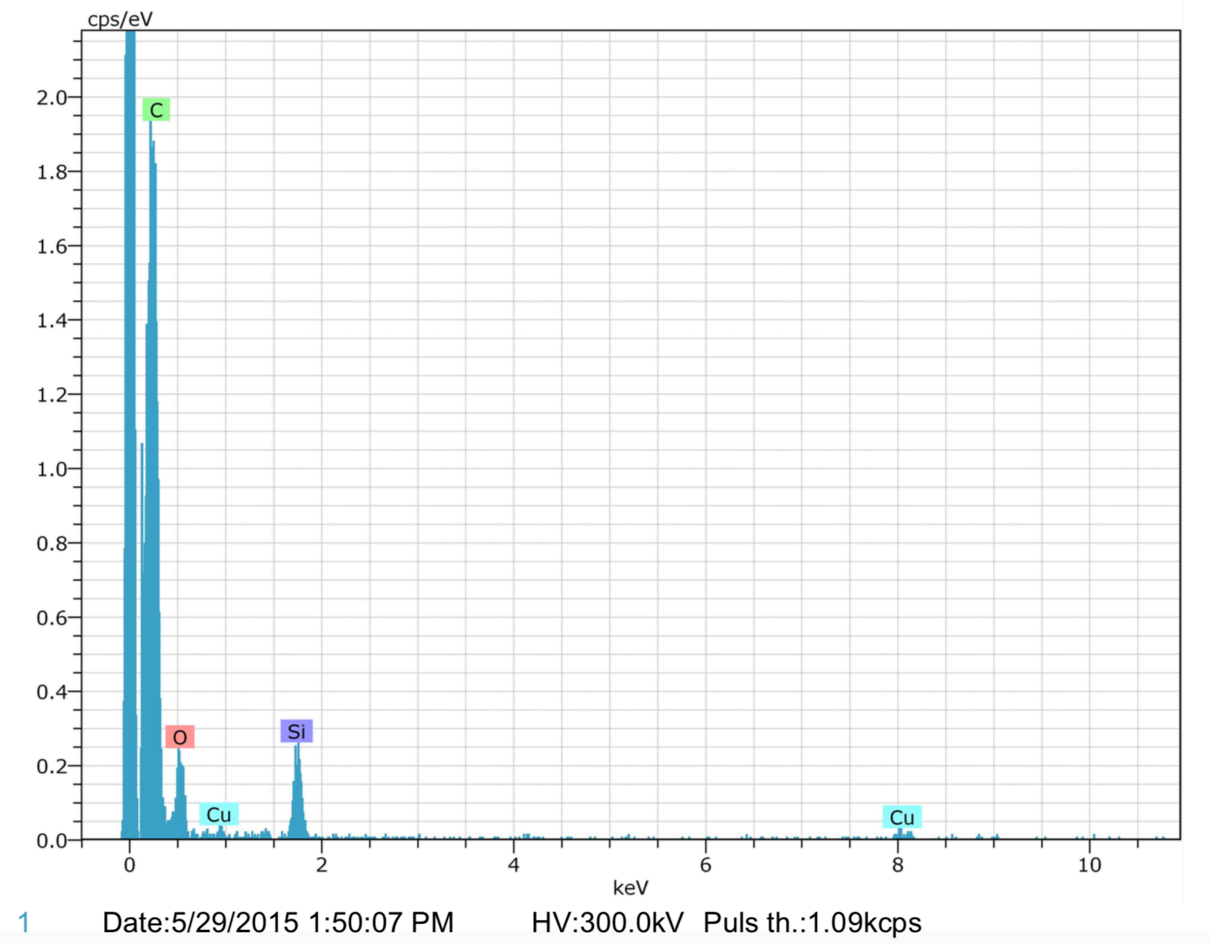

Supplementary Fig. 4. Spectrum showing the background composition in TEM-EDX spectra.
